# Severe changes in colon epithelium in the *Mecp2*-null mouse model of Rett syndrome

**DOI:** 10.1186/s40348-016-0065-3

**Published:** 2016-11-21

**Authors:** Pamela Millar-Büchner, Amber R. Philp, Noemí Gutierrez, Sandra Villanueva, Bredford Kerr, Carlos A. Flores

**Affiliations:** 1 Centro de Estudios Científicos (CECs), Avenida Arturo Prat 514, 511046 Valdivia, Chile; 2 Department of Translational Pulmonology, Translational Lung Research Center Heidelberg (TLRC), German Center for Lung Research (DZL), University of Heidelberg, Heidelberg, Germany; 3 Universidad Austral de Chile, Valdivia, Chile

## Abstract

**Background:**

Rett syndrome is best known due to its severe and devastating symptoms in the central nervous system. It is produced by mutations affecting the *Mecp2* gene that codes for a transcription factor. Nevertheless, evidence for MECP2 activity has been reported for tissues other than those of the central nervous system. Patients affected by Rett presented with intestinal affections whose origin is still not known. We have observed that the *Mecp2*-null mice presented with episodes of diarrhea, and decided to study the intestinal phenotype in these mice.

**Methods:**

*Mecp2*-null mice or bearing the conditional intestinal deletion of *MECP2* were used. Morphometirc and histologic analysis of intestine, and RT-PCR, western blot and immunodetection were perfomed on intestinal samples of the animals. Electrical parameters of the intestine were determined by Ussing chamber experiments in freshly isolated colon samples.

**Results:**

First we determined that MECP2 protein is mainly expressed in cells of the lower part of the colonic crypts and not in the small intestine. The colon of the *Mecp2*-null mice was shorter than that of the wild-type. Histological analysis showed that epithelial cells of the surface have abnormal localization of key membrane proteins like ClC-2 and NHE-3 that participate in the electroneutral NaCl absorption; nevertheless, electrogenic secretion and absorption remain unaltered. We also detected an increase in a proliferation marker in the crypts of the colon samples of the *Mecp2*-null mice, but the specific silencing of Mecp2 from intestinal epithelium was not able to recapitulate the intestinal phenotype of the *Mecp2*-null mice.

**Conclusions:**

In summary, we showed that the colon is severely affected by Mecp2 silencing in mice. Changes in colon length and epithelial histology are similar to those observed in colitis. Changes in the localization of proteins that participate in fluid absorption can explain watery stools, but the exclusive deletion of Mecp2 from the intestine did not reproduce colon changes observed in the *Mecp2*-null mice, indicating the participation of other cells in this phenotype and the complex interaction between different cell types in this disease.

**Electronic supplementary material:**

The online version of this article (doi:10.1186/s40348-016-0065-3) contains supplementary material, which is available to authorized users.

## Background

Rett syndrome (RTT) is a devastating neurodevelopmental disorder affecting mainly girls since their early childhood. The major cause of RTT are mutations affecting the methyl-CpG binding protein 2 (MECP2) coding gene [1]. MECP2 is a transcription factor with a dual role on gene expression. It was first described that MECP2 binds to methylated CpG dinucleotides, and by interacting with the histone deacetylase complex, compacts the chromatin and silences gene expression [2]. More recently, it was described that MECP2 is also able to induce the expression of some of its target genes, mechanism mediated by the interaction with the transcription factor Creb1 [3].

The most significant efforts to understand alterations produced by MECP2 deficiency have been concentrated in neurons and, more recently, in microglia and astrocytes [4–6]. Nevertheless, cellular abnormalities affecting other tissues such as the intestinal tract are unknown. RTT patients have a high incidence of symptoms affecting the lower section of the gastrointestinal tract like gastroparesis, chronic constipation, and gastric and intestinal perforations, mainly [7]. The first report of a RTT patient affected by colon cancer urges the understanding of the impact of MECP2 inactivation on epithelial cell function and intestinal physiology [8].

During the breeding of the *Mecp2*-null mice in our animal facility, we observed that these animals presented episodes of diarrhea at around 2 months of age. Therefore, we decided to study changes on intestinal physiology and histology as a result of genetic inactivation of the *Mecp2* gene in this RTT mouse model. Our analysis determined that *Mecp2*-null mice presented macroscopic abnormalities in the colon. Histological observation of intestinal samples allowed us to determine that the colon crypts are shorter in length and displayed altered histology of the surface epithelial cells. Immunohistochemical analysis showed that proteins of the electroneutral absorptive machinery were abnormally expressed in *Mecp2*-null mice, while electrogenic movement of ions was normal. Increased quantity of proliferating cells was observed in the colon epithelium of *Mecp2*-null mice. We were able to observe Mecp2 expression in epithelial cells from the lower part of the colon crypts, but the selective *Mecp2* knockdown in intestinal epithelium did not recapitulate the observed features of the *Mecp2-null* mice, suggesting a complex interaction between different tissues expressing Mecp2 that participates in the development of the multisystemic complaint including intestinal symptoms in RTT patients.

## Methods

### Animals

*Mecp2-null* colony founders were obtained from The Jackson Lab stock #003890 in a C57BL/6J:129/SvJ genetic background. Colony founders were outbred with C57BL/6 wild-type male for at least eight generations. To generate the conditional KO mice for *Mecp2* in the intestinal epithelium, we bred heterozygous Mecp2 female mice carrying one of the *Mecp2* allele flanked by loxP sites with the male mice carrying the Cre recombinase protein coding sequence under the control of Villin promoter. Animals were obtained from The Jackson Laboratory (Bar Harbor, ME, USA). The mice were bred in the C57BL/6J strain for at least eight generations. All mice were housed in ventilated racks under specific-pathogen-free conditions at a room temperature of 20 °C ± 2 °C in a 12/12 h light/dark cycle with food and water ad libitum. All animal procedures were reviewed and approved by the local Institutional Animal Care and Use Committee regulations. The animal facility of the Centro de Estudios Científicos (CECs) is accredited by AAALAC.

### Morphological and histology analysis

Adult mice were sacrificed by cervical dislocation. The small intestine and colon were removed, and the distance between the duodenum to ileum and the cecum to rectum, respectively, were measured. Tissues were fixed, embedded in paraffin and sliced into 4-μm-thick sections, followed by staining with hematoxylin and eosin (HE) or Periodic acid–Schiff (PAS). Digital images were captured with a light microscope (Olympus, CX31) attached to a computer using ×40 magnification and recorded using the MShot Digital Imaging systems program. The crypt length in the HE sections was measured (in ≥8 crypts per field) as the distance from the base to the apical side. The PAS sections were used in two different analysis: (1) counting PAS-positive cells per crypt, expressed as the percentage of PAS-positive cells normalized by the total number of epithelial cells lining the colonic crypt, and (2) PAS-positive pixels per micrometer, which was determined, converting the PAS-stained sections to grayscale and splitting it in the three-color channels (green, red, and blue). The threshold was adjusted to the green channel, which has the best contrast, to obtain the PAS-positive pixels. To normalize our results to the crypt longitude, three lines of known length (μm) were traced on each crypt and the PAS-positive pixels determined in this area. Both procedures were performed using the free software, ImageJ.

### Immunoblotting

To determine protein and gene expression in the intestine, section of the colon and small intestine from 8-week-old mice were rinsed with PBS and opened at the mesenteric border, and the epithelium was stripped of muscle using a glass slide. Tissues were homogenized in M-Per buffer by sonication using a protocol of 6 cycles of 10-s pulses at a 100% intensity followed by 10 s of rest using a QSONICA LLC sonicator (Model Q125, USA). Homogenates were centrifuged at 20,000×*g* at 4 °C in an eppendorf centrifuge (Model 5415R, Germany) for 30 min; the pellet was discarded and the proteins from the supernatant were quantified by Pierce BCA Protein Assay Kit (Thermo Scientific). Thirty micrograms of protein extract prepared in loading buffer was electrophoresed in denaturing polyacrylamide 4–12% gels (SDS-PAGE) in reducing conditions and transferred to PVDF membranes (Bio-Rad Laboratories, Hercules, CA, USA). Membranes were blocked in 0.05% non-fat milk and incubated with primary antibody against Mecp2 (Millipore 1:1000) and beta-actin (Santa Cruz 1:5000) for 2 h at 37 °C. After five washes for 10 min each in TBST, membranes were incubated with HRP-conjugated secondary antibody for 1 h at room temperature and then washed six times in TBST. The bands for Mecp2 and beta-actin were visualized by chemiluminescence Pierce West Femto (Life Technologies) and analyzed by exposing membranes in a C-Digit® Blot Scanner (Licor Model 3600, USA).

### Real-time qPCR

Ages of mice analyzed are given as days post conception (d.p.c), where the presence of vaginal plugs was considered as embryonic 0.5 d.p.c. The birth took place at 19 d.p.c (P0). Pregnant females (15.5 d.p.c and 18.5 d.p.c) were sacrificed by cervical dislocation. Embryos were dissected from the uteri and placed in PBS. Immediately following harvest, the colon was dissected and embedded in fresh Trizol Reagent (Invitrogen, CA, USA) and stored at −80 °C until RNA extraction. RNA was reverse-transcribed using the ImPrim-II^TM^ Reverse Transcription System (Promega) to synthesize single-stranded complementary DNA (cDNA) at a concentration of 2 μg/μL. PCR reaction mixtures were prepared using the KAPA SYBR FASTA qPCR kit. All amplification reactions were performed in triplicate using the Rotor Gene 6200, with a total volume of 10 μL, each reaction containing 1 μL of diluted cDNA. The real-time program used consisted in an initial denaturation period of 10 min at 95 °C followed by 40 cycles at 95 °C for 20 s, 58 °C for 15 s, and 72 °C for 30 s. The results were analyzed with the Rotor Gene-6000 series software 1.7 (Corbett) and all values were normalized to cyclophilin 1 messenger (RNA) mRNA expression levels. The primers used were the following: Mecp2 forward 5-CTCCATAAAAATACAGACTCACCAGT-3, Mecp2 reverse 5-CTTAAACTTCAGTGGCTTGTCT-3, cyclophilin forward 5-GGCAATGCTGGACCAAACACAA-3, and cyclophilin reverse 5-GTAAAATGCCCGCAAGTCAAAAG-3. Colon samples from adult animals were used for the quantification of mRNA for the ClC-2 accessory protein Glial-cam using the following primers forward: 5-GGGAGAAGACCATCAAC T-3 and reverse 5-TGAGCTCCAGCACAGTGGTT-3.

### RT-PCR

PCR reaction mixture was prepared in 20 μL total volume containing 2 μg of reverse-transcribed cDNA as described above. The amplification program consisted in an initial denaturation period of 10 min at 95 °C followed by 30 cycles at 95 °C for 20 s, 58 °C for 20 s, and 72 °C for 30 s. The primers used were the following: Mecp2e1 forward 5-AACGGGGTAGAAAGCCTG-3 and reverse 5-TGATGGGGTCCTCAGAGC-3 and for isoform, Mecp2e2 forward: 5-CAGGTCATGGTGATCAAACG-3 and reverse 5-AGTCCTTTCCCGCTCTTCTC-3. Cyclophilin 1 was used as loading control using the same primers described above. Amplicons were electrophoresed in a 1.5% acrylamide gel containing EtBr and visualized in an Ultraviolet GelDoc-it UVP Transiluminator photodocumenter.

### Immunohistochemistry

Colonic tissue was isolated and washed in phosphate buffer saline (PBS). Immunohistochemistry was performed as previously described [9]). Briefly, 4 mM paraffin sections were incubated with the corresponding primary antibody: ClC-2 1:400 (ACL-002, Alomone), NHE-3 1:1000 (C-20, Santa Cruz), and NKCC1 1:4000 (kindly provided by R. James Turner, [10]). To detect bound antibodies, we used the LSAB Universal Kit (Dako). The positive reaction was developed with diaminobenzidine or Vector SG (Vector Labs) and sections were counterstained with hematoxylin or nuclear fast red (Vector Labs).

For double immunohistochemistry, samples were first incubated with anti-ClC2 antibody, and peroxidase activity was visualized using Vector SG (Vector Labs). At the end of the first labeling reaction, sections were washed in 50 mM Tris HCl pH 7.8 and treated as described above for NHE3 immunolabeling. The counterstaining was performed with nuclear fast red (Vector Labs).

### Immunofluorescence

Colonic and small intestine paraffin-embedded sections (4 μm) were dewaxed and hydrated before the immunofluorescence procedure. Microwave antigen retrieval was performed in a 10-mM citrate pH 6. To permeabilize, the samples were incubated for 30 min with Triton 0.2% diluted in BSA 5% plus normal goat serum (NGS) 2.5%. Then, sections were incubated with anti-rabbit MECP2 antibody (1:100, Millipore) at room temperature for 2 h, washed with 50 mM Tris HCl pH 7.8, and incubated with secondary antibody Goat anti-Rabbit Alexa Fluor 488 (Life Technologies) for 30 min. The reaction was visualized in confocal microscopy (Olympus, BX61WI) and attached to Olympus Flouview FV 1000 software.

### Ussing chamber experiments

Experiments were performed as previously described [11]. Briefly, stripped colonic epithelium was placed in P2303 tissue holders of 0.1 cm^2^ surface and placed in modified Ussing chambers (Physiologic Instruments Inc., San Diego, CA, USA) bathed with bicarbonate-buffered solution (pH 7.4), gassed with 5% CO_2_–95% O_2_, and maintained throughout the experiment at 37 °C. The transepithelial potential difference referred to the serosal side was measured using a VCCMC2 amplifier (Physiologic Instruments Inc., San Diego, CA, USA). Current was clamped to 0 μA and 200 ms pulses of ±10 μA and was passed across the tissues at 1 s intervals using the Acquire & Analyze 2.3v software and a DI-720 interface (DataQ instruments, Akron, OH, USA).

### Plasmatic sodium

Blood was collected by retro-orbital plexus puncture from 60–62 days old animals. Sodium was determined using the EasyLyte analyzer (Medica Corporation, Bedford, MA, USA) under manufacturer’s instructions.

## Results

### Mecp2 is expressed in colonic epithelium

**Fig. 1:**
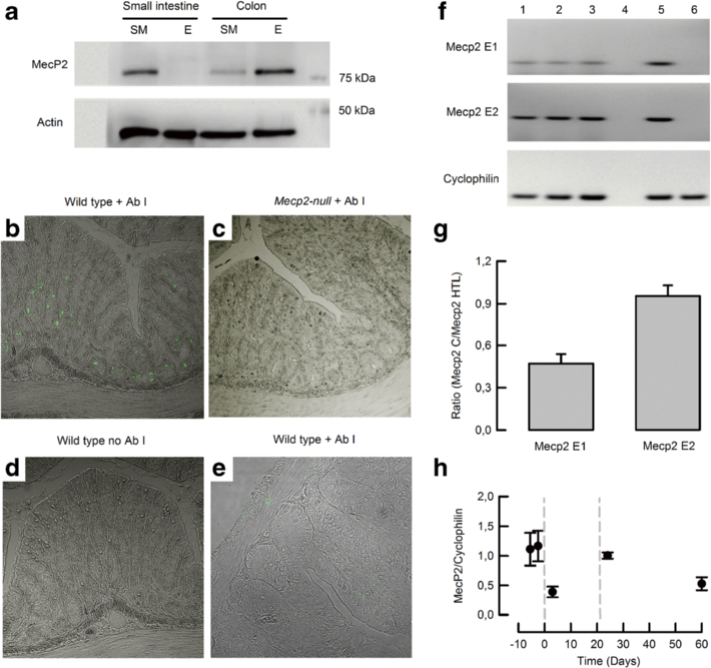
Protein and mRNA expression of Mecp2 in mouse intestine. **a** Representative western blot of Mecp2 from samples obtained from smooth muscle (SM) or epithelium (E) of the small intestine or colon of wild-type mice (*n* = 5). Mecp2 detection by immunofluorescence in **b** colon of wild-type, **c** colon of *Mecp2*-null mice, **d** colon of wild-type with omitted primary antibody, and **e** small intestine of wild-type mouse (*n* = 3 per group). **f** RT-PCR detection of Mecp2 variants E1 and E2 in colon epithelium. *Lanes 1–3* colon samples, *lane 4* RT-negative control, *lane 5* hypothalamus sample from a wild-type mouse, and *lane 6* hypothalamus sample from a *Mecp2*-null mouse. **g** Relative expression of Mecp2 variants on colon epithelium measured from the blots showed in **f** (*n* = 3). **h** qPCR from colon of embryonic 15.5 and 18.5 d.p.c. and 3, 24, and 60 days postpartum (*n* > 5 for each group)

Immunodetection of Mecp2 was performed in intestinal tissue of the mice. As shown in Fig. 1a Mecp2 was detected in both the colon and small intestine. Nevertheless, fractions of smooth muscle and epithelium demonstrate that Mecp2 is more abundant in the colon than in small intestine epithelium. To further determine tissue distribution of Mecp2, we performed immunofluorescense detection. Mecp2-positive cells were found mostly at the base of the colonic crypts (Fig. 1b), while no staining was detected in small intestine samples (Fig. 1e). The observed immunoreactivity was specific as no reaction was observed in colon samples from *Mecp2*-null mice (Fig. 1c) or wild-type mice when the primary antibody was omitted (Fig. 1d).

To determine which Mecp2 variants are expressed in the colon epithelium, we performed PCR detection of Mecp2-E1 and Mecp2-E2. As shown in Fig. 1F, colon tissue expressed both variants, but when compared to hypothalamus expression, colon tissues preferentially expressed Mecp2-E2 (Fig. 1g). Finally, real-time PCR experiments showed that Mecp2 was highly expressed at the end of fetal development, changing expression levels with time, decreasing after birth and increasing almost 2 times after weaning (day 21), and maintaining relatively high levels of expression up to 60 days of age (Fig. 1h).

### The *Mecp2*-null mice exhibit altered colon morphology and histology

**Fig. 2:**
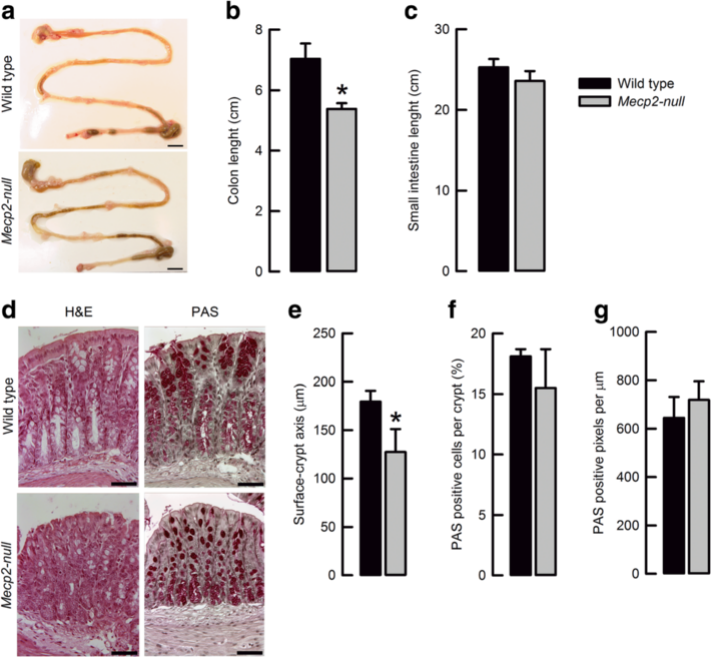
Mecp2 deletion produces changes on colon morphology and histology. **a** Representative images of intestinal morphology (bar 1 mm). **b** Summary of average colon and **c** small intestine length from wild-type and *Mecp2*-null mice.*P < 0.05 vs. wild-type (*n* = 6 each genotype). **d** Representative images of histology H&E staining (*upper*) and PAS staining (*lower*) (bar 25 μm). **e** Surface–crypt axis measurements of wild-type and Mecp2-null colon samples. *P < 0.05 vs. wild-type (*n* = 6 for each genotype). **f** PAS-positive cells and **g** PAS-positive pixels in wild-type and Mecp2-null colon samples (*n* = 6 for each genotype)

Observation of intestinal anatomical features of 8 weeks old *Mecp2*-null mice suggested changes in colon size (Fig. 2a). Measurement of intestine sections showed that the colon (Fig. 2b) but not the small intestine (Fig. 2c) was shortened in the *Mecp2-null* mice.

Histology of distal colon samples showed that in some areas, organization of cells of the surface seems to be altered (Fig. 2d). The surface–crypt axis appeared smaller in the *Mecp2*-null mice (Fig. 2d), an observation corroborated by direct measurements (Fig. 2e). The number of goblet cells (Fig. 2f) and content of PAS-positive pixels (Fig. 2g) was unaltered by the absence of Mecp2 in the colon. The described alterations seemed to be age dependent, since 4-week-old animals showed unaltered colon and crypt length when compared to wild-type littermates (data not shown). Histology of small intestine appeared normal.

### Colon from Mecp2-null mice showed aberrant expression of ClC-2 and NHE-3 proteins

**Fig. 3:**
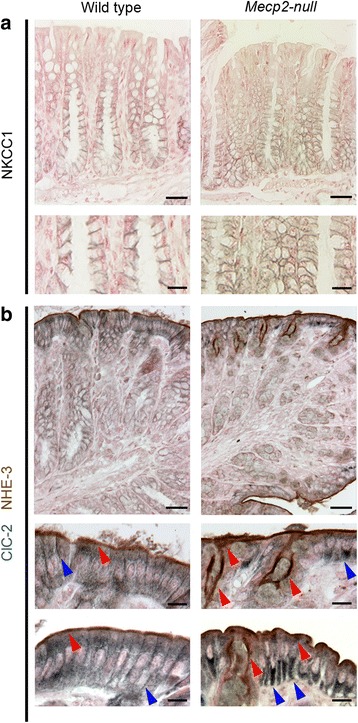
Comparison of NKCC1, ClC-2, and NHE-3 distribution in mouse colon. **a** Immunoperoxidase labeling for NKCC1 in the distal colon of wild-type (*left panels*) and *Mecp2*-null (*right panels*) animals. **b** Double immunoperoxidase labeling for ClC-2 (*blue arrows*) and NHE-3 (*red arrows*) in distal colon samples. Representative images of samples taken from five mice of each genotype. *Bars A and B upper* 25 μm and *A and B lower* 10 μm

In order to obtain a more detailed view of the organization of the different cell types that compose colon epithelium, we performed immunohistochemical detection of NKCC1 and ClC-2 membrane proteins to differentiate and localize epithelial cells of the crypt base and surface, respectively [9]. We observed that the NKCC1 triple cotransporter was localized in the basolateral membrane of lower and medial parts of the crypts in both wild-type and *Mecp2*-null colon (Fig. 3a). ClC-2, known to localize in the basolateral membrane of the surface cells, was accompanied by NHE-3 detection, the interchanger localized at the apical membrane of the surface cells. As can be observed in Fig. 3b, distribution of ClC-2 and NHE-3 is altered in most areas of the distal colon of the *Mecp2*-null animal. ClC-2 was barely detected in the basolateral membrane and was retained in the cytoplasm of the surface cells. NHE-3 was mostly retained at the apical membrane of surface cells, nevertheless, some cells showed intracellular staining and NHE-3-positive staining is also observed in cells in the entry of the crypts (Fig. 3b, right panels).

Glial-cam is an accessory protein known to interact with ClC-2 and target the channel to cell-cell junctions [12]. To determine if Glial-cam expression is altered and could explain ClC-2 intracellular retention in *Mecp2*-null colon, we determined its mRNA expression level. Real-time PCR studies showed no differences in Glial-cam mRNA expression between wild-type and *Mecp2*-null colon (data not shown). Thus, increased intracellular localization of ClC-2 is not associated to changes in Glial-cam mRNA expression.

### Ussing chamber measurements showed no differences in absorption and secretion in *Mecp2*-null mice colon

**Fig. 4:**
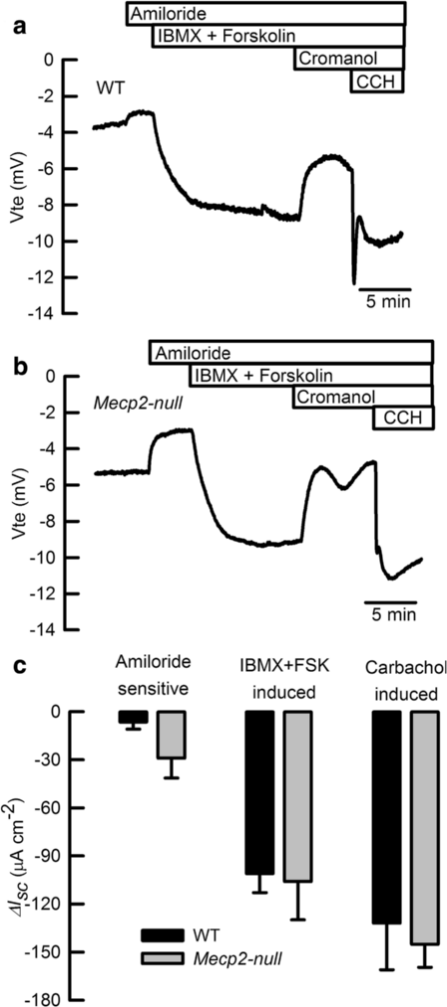
Recorded *V*_*te*_ traces of **a** wild-type and **b** *Mecp2*-null colon samples. **c** summarizes equivalent Isc calculation for amiloride-sensitive sodium absorption, cAMP-dependent anionic secretion (IBMX + FSK-induced) and calcium-activated anionic secretion (carbachol-induced). *n* = 5 for each group

To determine if the observed cellular alterations in *Mecp2*-null mice impacts electrogenic absorption and secretion of ions in colon, we performed Ussing chamber measurements of the colon tissues dissected from wild-type and *Mecp2*-null mice. We observed no differences in basal electrical parameters between both genotypes obtained from experiments like those shown in Fig. 4a, b. Calculated values were as follows: *V*_*te*_ −3.7 ± 1.2 v/s −5.8 ± 1.2; *I*_*sc*_ −63 ± 13 v/s −64 ± 16, and *R*_*te*_ 75 ± 8 v/s 63 ± 4 for wild-type and *Mecp2*-null mice, respectively. Calculation of short-circuit currents for electrogenic cAMP-dependent (IBMX + FSK-induced) or calcium-dependent (carbachol-induced) chloride secretion showed no significant differences among groups. A slight increase in electrogenic sodium absorption (amiloride-sensitive) was observed in the *Mecp2*-null colon (summarized in Fig. 4c), and without affecting systemic sodium homeostasis (149 ± 3 mM v/s 148 ± 4 mM sodium in plasma for wild-type (*n* = 5) and *Mecp2* null (*n* = 3), respectively).

### Conditional deletion of *Mecp2* from intestinal tissue does not reproduce the *Mecp2*-null mouse phenotype

**Fig. 5:**
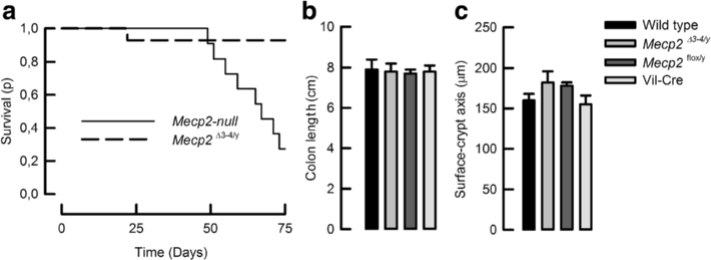
Intestinal deletion of Mecp2 does not replicate *Mecp2*-null intestinal phenotype. **a** A group of 14 *Mecp2*-null animals (*solid line*) was compared with a group of 11 *Mecp2* ^Δ3–4/y^ conditional knock-out mice (*dashed line*). Kolmogorov–Smirnov test demonstrated a better survival rate for the conditional knock-out animals over the *Mecp2*-null mice, *P* < 0.0005. Determinations of **b** colon length and **c** surface–crypt axis length showed no differences among groups (*n* = 5 for wild-type, *n* = 9 for *Mecp2* ^Δ3–4/y^, *n* = 4 for *Mecp2* ^flox/y^, and *n* = 6 for Vil-Cre)

To study if Mecp2 silencing in intestinal epithelium was able to reproduce the phenotype observed in *Mecp2*-null mice, transgenic animals expressing Cre recombinase under the control of the Villin promoter (Vil-cre) were crossed with *Mecp2* ^*flox/y*^ female mice to induce the deletion of loxP-flanked exon 3 and 4 of *Mecp2*. Silencing of *Mecp2* from colon epithelium was checked by immuno detection (Additional file 1: Fig S1). As shown in Fig. 5a, the *Mecp2* ^*Δ3–4/Y*^ mice presented significantly reduced lethality compared to *Mecp2*-null animals. Analysis of intestinal tissues showed that the lengths of the colon (Fig. 5b) and small intestine (data not shown) were not altered, nor the colon crypt length (Fig. 5c) in the *Mecp2* ^*Δ3–4/Y*^ mice.

**Fig. 6:**
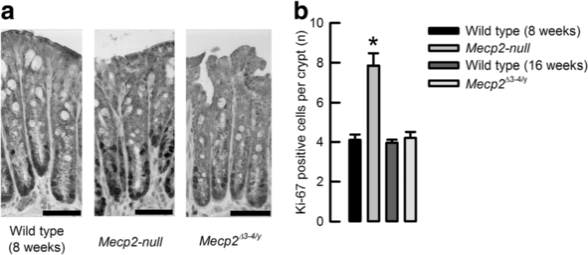
Ki-67-positive cells are increased in the Mecp2-null animals. **a** Representative images of Ki-67 immunodetection in colon samples (bar 25 μm). **b** Summary of Ki-67-positive cells per crypt for wild-type 8 weeks old (*n* = 3), Mecp2-null 8 weeks old (*n* = 3), wild-type 16 weeks old (*n* = 5), and *Mecp2* ^Δ3–4/y^ 16 weeks old (*n* = 4). *P < 0.03 compared to wild-type 8 weeks old

Shortening of colon and surface–crypt lengths are normally related to the changes in the proliferation rate of intestinal cells. We studied the expression of Ki-67 in colon samples and observed that Mecp2-null colon exhibited a larger number of Ki-67-positive cells than control tissues. Samples of *Mecp2* ^*Δ3–4/Y*^ colon showed a number of Ki-67-positive cells comparable to those of control tissues (Fig. 6b). In all cases, Ki-67-positive cells were situated at the crypt base (Fig. 6a).

## Discussion

Rett syndrome is a genetic disorder that severely affects normal development of the CNS. Nevertheless, there is evidence for symptoms affecting peripheral tissues, and the efforts to understand the impact of *MECP2* mutations in the function of other systems in the organism have recently led to more detailed descriptions of phenotypes affecting the respiratory, cardiovascular, and immune systems [4, 6, 13, 14]. Symptoms affecting the gastrointestinal tract of RTT patients are common, but how the absence of MECP2 impacts intestinal function is currently unknown. We observed that *Mecp2* mRNA expression in the colon decreases after birth and increases after weaning on day 21 up to day 60, mimicking what has been observed in the brain [15]. It has been reported that the major Mecp2 isoform expressed in the postnatal brain is Mecp2-E1 [16]; however, our analysis showed that the main isoform expressed in colon tissue of adult animals corresponds to Mecp2-E2. Even though some functional differences among isoforms have been proposed, this has been partially examined in neurons exclusively where the absence of Mecp2-E2 protects cultured cerebellar granule neurons from neurotoxicity [17], but nothing is known about what is the impact of the isoform deletion in other tissues since Mecp2-E2 silencing compromises embryo viability [18].

Examining Mecp2 distribution in intestinal tissues of adult mice revealed some differences between immunofluorescence (colon epithelial cells exclusively) and western blot (colon epithelium and, occasionally, smooth muscles from the colon and small intestine). The strongest signal observed by western blot was detected in colon epithelium correlating with the immunofluorescence localization. The faint signal might be due to contamination of smooth muscle samples with epithelial cells after mechanical stripping, or the presence of Mecp2 expressing cell types like those of the enteric nervous system or macrophages that can be present in the smooth muscle layer [4, 19]. MECP2 expression in the intestine has been previously documented in human, rat, and mouse tissues, and the different results obtained might reflect not only species differences but also handling of samples and heterogeneity of technics used for MECP2 detection. For example, western blot analysis of adult mouse tissues showed Mecp2 expression in the colon [20], and the same authors detected northern blot signal for Mecp2 in the colon but not in the small intestine from humans. Nevertheless, expression of Mecp2 mRNA has been detected in epithelial cells of the small intestine of adult rats [21], and other immunodetection studies of MECP2 distribution determined that the protein is expressed in the smooth muscle [22] and in cells of the enteric nervous system of the mouse intestine [19]. Our immunofluorescence experiments showed no signal for MECP2 on the smooth muscle layer. We tested the specificity of the antibody in the colon tissue from the *Mecp2*-null mouse and no signal was detected in both the mucosa and smooth muscle.

Morphological analysis of the intestine showed a significant reduction of the colon and crypt length in *Mecp2*-null mice. Colon and crypt shortening has been observed in animal models of colitis and inflammatory bowel disease, as well as in mice when dyslipidemia was induced with a high-fat diet [23–26]. Dyslipidemia has been observed in the *Mecp2* ^−/−^ animals and in a significant number of Rett patients [27]. Interestingly, *Mecp2* ^*−*/−^ animals subjected to pharmacotherapy to improve lipid metabolism showed amelioration of motor symptoms and decreased lethality [28], but the possibility that the pharmacological restoration of lipid homeostasis is capable to ameliorate the intestinal phenotype in the *Mecp2* ^−/−^ animals has not been explored.

Our results indicate that MECP2 is expressed in colon epithelial cells, and more specifically in the lower section of the crypts where the stem cell niche and cells with fluid secretion capacity are housed. We observed that absence of MECP2 produces shortening of surface–crypt axis lengths. Nevertheless, we observed histological changes in cells from the surface and not in the crypt where Mecp2 was localized. In fact, the localization and distribution of a main membrane component of electrolyte movement in the colon, the triple cotransporter NKCC1, appears to be normal in cells of the crypt in *Mecp2* ^−/−^ colon. The latter observation is supported by Ussing chamber experiments that showed intact electrogenic anionic secretion in these animals. The *Mecp2*-null mouse showed aberrant expression of membrane proteins such as the chloride channel ClC-2 and the sodium–proton exchanger NHE3 both located in cells at the crypt surface and major players of electroneutral absorption of electrolytes in the colon. As we have previously demonstrated, the absence of ClC-2 produces a severe reduction of NaCl absorption [29], and *Scl9a3* ^−/−^ (*Nhe3-null*) mice presented with slight diarrhea due to reduced sodium absorption in the colon [30]. Moreover, increased electrogenic sodium absorption via ENaC has been described in both *Clc2* ^*−*/−^ and *Scl9a3* ^−/−^ mouse colon as a compensatory mechanism for reduced electrolyte absorption. Our own examination of electrogenic movement of electrolytes in colon showed a tendency to increase amiloride-sensitive currents (reflecting ENaC-dependent sodium absorption) in the *Mecp2*-null colon that did not reached statistical significance, but that might reflect defective electroneutral NaCl absorption. Nevertheless, plasmatic sodium is unaffected indicating that sodium homeostasis is normal in the *Mecp2*-null mice and that the intestinal malfunction might be triggered at a very late stage of the disease and for a short period of time that is not long enough to reflect sodium reduction in the circulation. It is also important to comment that a significantly increased intestinal transit time has been recently reported to occur in the *Mecp2*-null mice and that could also favor malabsorption and the appearance of watery stools observed by us in the *Mecp2*-null animals [31].

After we described the effects of MECP2 absence in the colon, we questioned if using the Cre-loxP system the sole deletion of *Mecp2* from intestinal epithelium was sufficient to reproduce the intestinal phenotype of the *Mecp2*-null mouse. We first observed that intestinal deletion of *Mecp2* did not bear a lethal phenotype and, most surprisingly, it did not reproduce the colon and surface–crypt axis length changes as the *Mecp2*-null mouse. Such absence of changes in colon morphology and histology was sustained up to 16 weeks of life.

We observed an increase of the proliferation marker protein Ki-67 in colon epithelial cells of *Mecp2*-null animals, a similar finding that has been reported in colitis models involving mice [32, 33]. Even though cells expressing Ki-67 expression are found at the same location where we detected MECP2 in the cells of the lower part of the colon crypts, the exclusive silencing of *Mecp2* from the intestine does not increase the number of Ki-67-positive cells, suggesting that Rett phenotype in colon can be originated by factors released outside the colon or by altered activity of cells other than colonic epithelium like the enteric nervous system where MECP2 is expressed [31]. Other explanation might be that cells in the surface can be affected by alterations in the intestinal contents delivered from the small intestine into the colon. For example, bile acids are known to induce colitis in mice and humans [34, 35], and even when there is no available data for fecal bile acid contents in RTT patients or animal models, scattered reports of RTT patients hospitalized due to liver failure and many others subjected to gallbladder removal are available [27]. More recently, it has been published that Rett patients presented with a less diverse microbiota than healthy subjects [36], which could affect gastrointestinal function, but this possibility has not been explored in the mouse model yet.

## Conclusions

In summary, we have described for the first time the effects of *Mecp2* silencing on mouse intestine. Our results indicate that the absence of Mecp2 induces shortening of the colon length and depth of crypts. Along with histological changes in the cells at the surface of the colon and mislocalization of key membrane proteins responsible for electroneutral fluid absorption, this might favor fluid accumulation often seen in the *Mecp2*-null mice. Importantly, the sole silencing of *Mecp2* from intestinal epithelium did not account for the complete phenotype, suggesting the participation of other cells that express MECP2 and regulate intestinal function as well as and the profound complexity of this disease.

## Additional file

Additional file 1: Figure S1. Intestinal MECP2 deletion using the villin-Cre Tg mouse. Immunofluorescence was performed to detect MECP2 protein in colon samples from (A) *Mecp2* ^flox/y^ mouse and (B) *Mecp2* ^Δ3–4/y^. Positive signal was detected in the *Mecp2* ^Δ3–4/y^ mouse at the bottom of the crypts mainly. Representative images of three animals per group. (DOCX 211 kb)
