# Supplementary material for: Severe changes in colon epithelium in the Mecp2-null mouse model of Rett syndrome
Source: Mol Cell Pediatr. 2016 Nov 21;3:37. doi: 10.1186/s40348-016-0065-3 (PMC5116442; doi:10.1186/s40348-016-0065-3)
Supplement: Additional file 1: Figure S1. — Intestinal MECP2 deletion using the villin-Cre Tg mouse. Immunofluorescence was performed to detect MECP2 protein in colon samples from (A) Mecp2 flox/y mouse and (B) Mecp2 Δ3–4/y. Positive signal was detected in the Mecp2 Δ3–4/y mouse at the bottom of the crypts mainly. Representative images of three animals per group. (DOCX 211 kb) [file 40348_2016_65_MOESM1_ESM.docx]

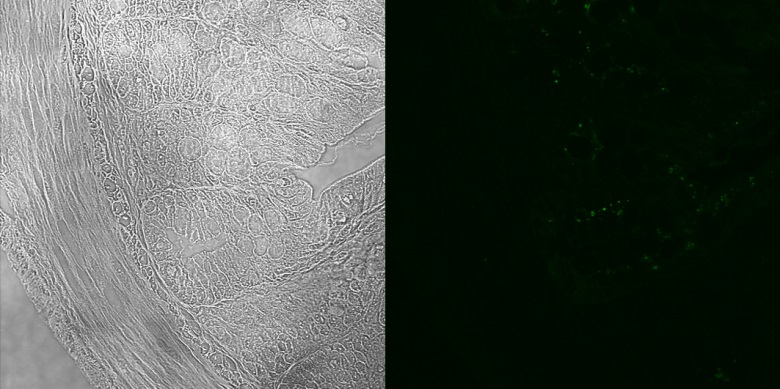
A



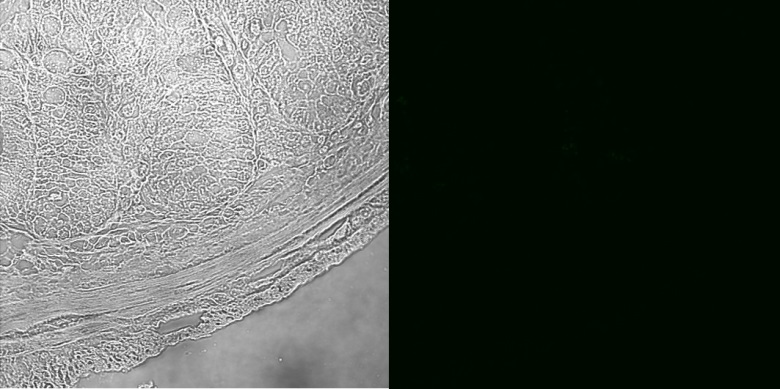
B

**Supplemental Figure 1. Intestinal MECP2 deletion using the villin-Cre Tg mouse.** Immunofluorescence was performed to detect MECP2 protein in colon samples from (A) *Mecp2*^flox/y^ mouse and (B) *Mecp2*^Δ3-4/y^. Positive signal was detected in the *Mecp2*^Δ3-4/y^ mouse at the bottom of the crypts mainly. Representative images of 3 animals per group.
